# Costunolide induces mitochondria-mediated apoptosis in human gastric adenocarcinoma BGC-823 cells

**DOI:** 10.1186/s12906-019-2569-6

**Published:** 2019-06-26

**Authors:** Zhanpeng Yan, Tingting Xu, Zhentao An, Ying Hu, Wanzhen Chen, Jinxia Ma, Changle Shao, Fangshi Zhu

**Affiliations:** 1 Affiliated Hospital of Integrated Traditional Chinese and Western Medicine, Nanjing University of Chinese Medicine, Nanjing, 210028 Jiangsu China; 2 Clinical Research Department of Chinese and Western Medicine, Jiangsu Province Institute of Traditional Chinese Medicine, Nanjing, 210028 Jiangsu China

**Keywords:** Costunolide, Apoptosis, Gastric cancer, Xenografted nude mice

## Abstract

**Background:**

Costunolide, a sesquiterpene lactone extracted from *Radix Aucklandiae*, has the activity against multiple cancers. However, the effect of costunolide on gastric cancer (GC) have remained to be ambiguous. In this study, we investigated the underlying mechanisms of apoptosis induced by costunolide in human gastric adenocarcinoma BGC-823 cells in vitro and in vivo.

**Methods:**

The viability of BGC-823 cells was detected by MTT assay. The apoptosis and mitochondrial membrane potential (ΔΨm) of BGC-823 cells induced by costunolide were analyzed by flow cytometry. The inhibiton of costunolide on human gastric adenocarcinoma was estimated in xenografts in nude mice. Apoptosis related proteins and genes were detected by Western blot and Q-PCR.

**Results:**

Costunolide inhibited the viability of BGC-823 cells in a time and concentration dependent manner. Costunolide induced the apoptosis and lowered the ΔΨm of BGC-823 cells significantly. Costunolide increased the expression of Bax, cleaved caspase 9, cleaved caspase 7, cleaved caspase 3 and cleaved poly ADP ribose polymerase (PARP) proteins and decreased the expression of Bcl-2, pro-caspase 9, pro-caspase 7, pro-caspase 3 and PARP proteins. Costunolide upregulated the expression of puma, Bak1 and Bax mRNA and downregulated the expression of Bcl-2 mRNA. In addition, we demonstrated that costunolide inhibited the growth and induced apoptosis of BGC-823 cells xenografted in athymic nude mice. Costunolide increased the expression of cleaved caspase 9, cleaved caspase 3 and Bax proteins and decreased the expression of Bcl-2 protein in xenografted tumor. Costunolide upregulated the expression of puma and Bax mRNA and decreased the expression of Bcl-2 mRNA in xenografted tumor.

**Conclusions:**

Collectively, our results suggested that costunolide induced mitochondria-mediated apoptosis in human gastric adenocarcinoma BGC-823 cells and could be the candidate drug against GC in clinical practice.

## Background

Gastric cancer (GC) is the common malignancies of digest system and the third leading cause of cancer-related deaths all over the world [1, 2]. The pathogenesis of GC is a complex and long-time multistep process, which is closely related to abnormal expression of many genes. The treatment for GC contains surgery, chemotherapy, radiation therapy and so on [3, 4]. Although the new treatment for GC has developed greatly in recent years, the therapeutic effect and prognosis of GC is still unsatisfactory [1, 4]. Therefore, it’s necessary and urgent to seek the effective treatment for GC.

Apoptosis is a process of programmed cell death and plays an important role in regulation of organ development and tissue carcinogenesis [5]. The pathogenesis of GC is closely related to abnormal apoptosis of gastric gland cells. Previous reports showed that inducing apoptosis of cancer cells is the critical method of treatment of GC [6]. Therefore, discovering the new effective drug against GC and researching the underlying mechanisms are required and valued via the perspective of apoptosis.

Costunolide, also called costus lactone, is a sesquiterpene lactone extracted from *Radix Aucklandiae* and exhibits the properties of anti-cancer [7]. The plant *Radix Aucklandiae* of the family Compositae is a common drug of traditional Chinese medicine. In ancient times, people thought the root of this plant has the function of promoting the circulation of blood and relieving pain [8]. It was reported that costunolide could inhibits proliferation, arrests cell cycle and induces apoptosis of many cancers, such as breast cancer, colon cancer, bladder cancer, prostate cancer and hepatocellular carcinoma cells [9–17]. However, the research on GC inhibited by costunolide is relatively few. Therefore, in this study, the therapeutic effect of costunolide on GC and the mechanisms have been explored in vivo and in vitro. This research will provide the experimental evidence for GC treated by costunolide.

## Methods

### Reagents

**Fig. 1:**
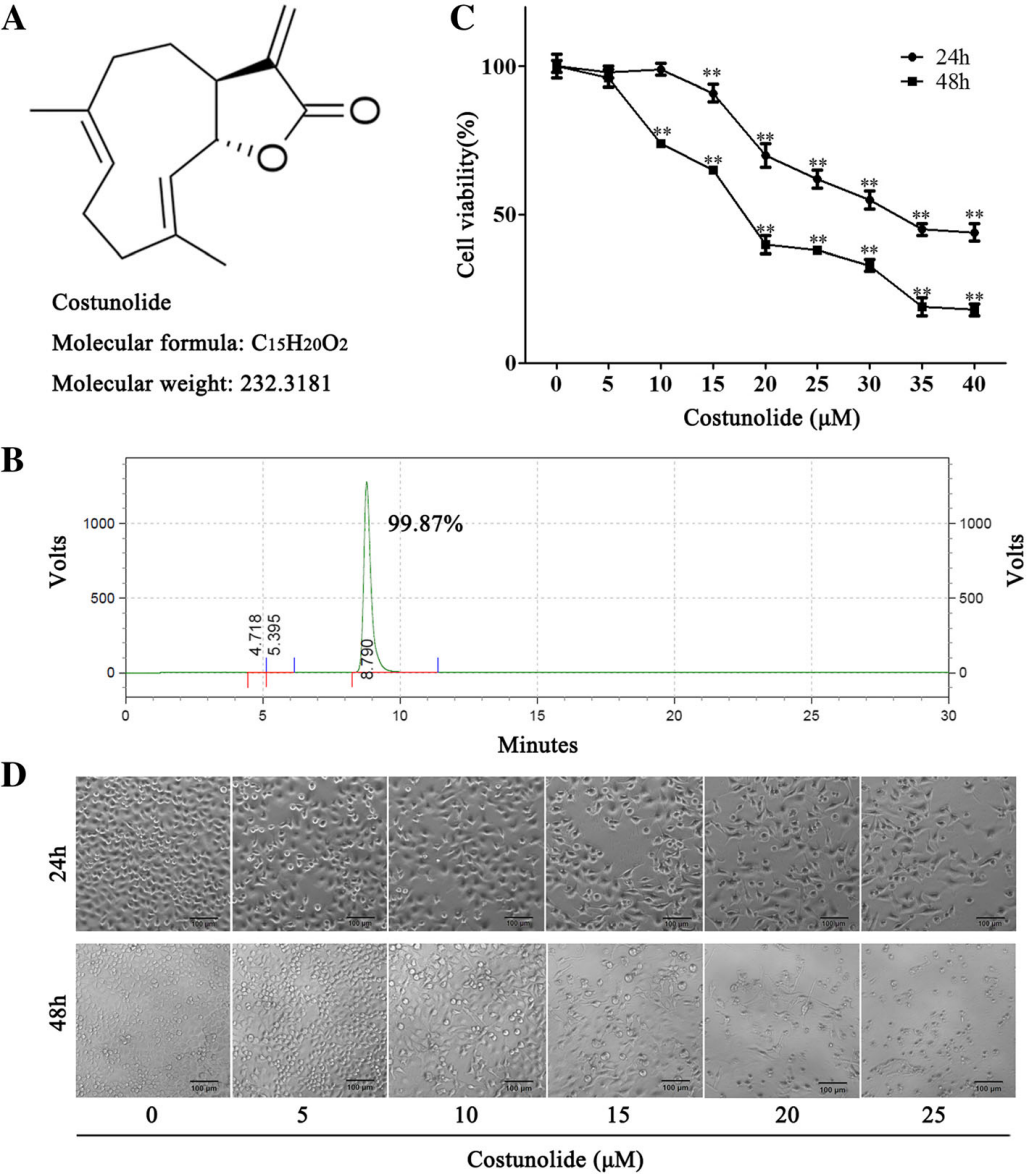
Costunolide inhibited the viability of gastric adenocarcinoma BGC-823 cells. **a** Chemical structure of costunolide. Molecular formula: C_15_H_20_O_2_, molecular weight: 232.32, CAS No.: 553–21-9. **b** Purity of costunolide detected by HPLC. The purity is 99.87%. **c** The viability of BGC-823 cells treated by costunolide with different concentration (0, 5, 10, 15, 20, 25, 30, 35 and 40 μM) for 24 and 48 h is detected by MTT assay. **d** The morphology of BGC-823 cells treated by costunolide with different concentration (0, 5, 10, 15, 20 and 25 μM) for 24 and 48 h is observed by light inverted microscopy (magnification: 100×, bar in the picture represents 100 μm). Compared to control group, *P < 0.05, **P < 0.01

Costunolide (Fig. 1a) was purchased from Chendu Must Biological Technology Limited Campany (Chendu, China). RPMI 1640 medium, fetal bovine serum (FBS), Trypsin-EDTA (0.25%), TRIzol reagent, Reverse Transcription PCR kit and Quantitative-PCR kit were obtained from Thermo Fisher Scientific (Massachusetts, USA). Tunel staining kit, Hoechst33258 staining kit, JC-1 staining kit and MTT were purchased from Shanghai Beyotime Biotechnology Co., Ltd. (Shanghai, China). Annexin V-FITC apoptosis detection kit was purchased from BD Biosciences (California, USA). Rabbit anti-human Bax, Bcl-2, caspase 9, caspase 7, caspase 3, cleaved caspase 3, PARP and β-actin antibodies were purchased from Cell Signaling Technology (Massachusetts, USA). Dylight 800-labeled goat anti-rabbit IgG fluorescence antibody was purchased from KPL (Massachusetts, USA).

### Cell line and cell culture

The human gastric adenocarcinoma BGC-823 cell line was obtained from Nanjing University of Traditional Chinese Medical. After the cells were recovered, cells were used as experiments from the third generation. Cells were cultured in RPMI 1640 medium supplemented with 10% FBS, 100 U/mL penicillin and 100 μg/mL streptomycin. Cells were maintained in saturated humidity cell incubator (Thermo Fisher Scientific, Massachusetts, USA) with 5% CO_2_ at 37 °C.

### Nude mice

Sixteen female athymic BALB/c nude mice (body weight is 20 ± 2 g, age is 4~6 weeks old) were purchased from Shanghai SLAC Laboratory Animal Co., Ltd. (Shanghai, China). The animal license No. is SCXK 2017–0005. Mice were housed in specific pathogen free (SPF) laboratory animal environment in Animal Center of Jiangsu Province Institute of Traditional Chinese Medicine. Animal feeding meets the requirements of guidelines for the housing of mice in scientific institutions. There are four nude mice in each cage. The nude mice eat and drink freely and were exposed to a day:night cycle of 12 h of day and 12 h of night. The method of euthanasia in mice is cervical dislocation. Animal experiments of this study were submitted to and approved by Ethics Committee of Jiangsu Province Hospital on Integration of Chinese and Western Medicine. All animal experiments were conducted according to the Guideline of Welfare and Ethics of Laboratory Animals (issued by the General Administration of Quality Supervision, Inspection and Quarantine of the People’s Republic of China) and the Guide for the Care and Use of Laboratory Animals (issued by the US National Institutes of Health).

### High-performance liquid chromatography (HPLC)

Costunolide solution (0.4 mg/mL) is prepared in 80% ethanol. Costunolide was detected by HPLC Agilent 1100 series (Agilent Technologies, Inc., California, USA). Chromatographic column: analytical column (particle size: 5 μm) COSMOSIL 5C_18_-PAQ, 250 mm × 4.6 mm (Nacalai Tesque, Inc., Kyoto, Japan). Column temperature is 30 °C. Mobile phrase varies as below: from 75% ethanol to 75% ethanol in 0–30 min. Flow rate is 0.8 mL/min. Detection wavelength is 254 nm. Sample loading volume is 5 μL.

### Cell viability assay and observation of cell morphology

BGC-823 cells were seeded in 96-well plates at the density of 10^4^ cells/well in 100 μL medium. After cells were cultured to 60~70% confluence/well, cells were treated by costunolide with different concentration (0, 5, 10, 15, 20, 25, 30, 35 and 40 μM) for 24 h. Cells was observed and photographed by using inverted light microscopy (magnification: 40×) (Olympus, Japan). Then 10 μL MTT solution (5 mg/mL) was added to wells and cells were cultured for 4 h. Medium was removed and 100 μL DMSO was added to each wells. The optical density (OD) was measured at absorbance wavelength 570 nm using the Full-wavelength Microplate Reader (Tecan Group, Ltd., Mannedorf, Switzerland). Cell viability was calculated as a proportion by using the following formula: Cell viability (%) = OD_570nm_ (treated)/OD_570nm_ (untreated) × 100%.

### Hoechst33258 staining

Coverslip was placed into the well of cell culture plate. BGC-823 cells were seeded in 6-well plates at the density of 3 × 10^5^ cells/well in 2 mL medium. After cells were cultured to 60~70% confluence/well, cells were treated by costunolide with different concentration (0, 5, 10, 15, 20 and 25 μM) for 24 h. Cells were fixed with 0.5 mL Fixed solution for 10 min and incubated with Hoechst33258. Anti-fluorescence quenching liquid was droped in the slide and the coverslip was covered on slide. Cells were observed by fluorescent inverted microscope (Olympus, Tokyo, Japan).

### Flow cytometry analysis of apoptosis

BGC-823 cells were seeded in 6-well plates at the density of 3 × 10^5^ cells/well in 2 mL medium. After cells were cultured to 60~70% confluence/well, cells were treated by costunolide with different concentration (0, 5, 10, 15, 20 and 25 μM) for 24 h. Cells were digested by using Trypsin and collected in tube. Cells were stained with 5 μL propidium iodide (PI) and 5 μL Annexin V-FITC and incubated for 15 min at room temperature in the dark. Cells were detected by flow cytometer (Merck Millipore, Darmstadt, Germany). The date of flow cytometry was analyzed by the guavaSoft software (Merck Millipore, Darmstadt, Germany).

### Flow cytometry analysis of ΔΨm

BGC-823 cells were seeded in 6-well plates at the density of 3 × 10^5^ cells/well in 2 mL medium. After cells were cultured to 60~70% confluence/well, cells were treated by costunolide with different concentration (0, 5, 10, 15, 20 and 25 μM) for 24 h. Cells were digested by using Trypsin and collected in tube. Cells were resuspended with 0.5 mL medium and 0.5 mL JC-1 staining solution for 20 min at 37 °C. Cells were detected by flow cytometer (Merck Millipore, Darmstadt, Germany). The ΔΨm was analyzed by the guavaSoft software (Merck Millipore, Darmstadt, Germany).

### Western blot (WB)

BGC-823 cells were seeded in 10 cm culture dish at the density of 2 × 10^6^ cells/dish in 10 mL medium. Cells were treated by costunolide with different concentration (0, 5, 10, 15, 20 and 25 μM) for 24 h. Cells were treated RIPA lysis buffer containing protease and phosphatase inhibitor for 10 min. Total protein was collected by centrifuging (12,000×g, 10 min, 4 °C) and mixed with the same volume of sample buffer. Extracted protein was separated using sodium dodecyl sulfate polyacrylamide gel electrophoresis (SDS-PAGE) and transferred to the polyvinylidene difluoride (PVDF) membrane. PVDF membrane was blocked and incubated with rabbit anti-human primary antibody (Bax, Bcl-2, caspase 9, caspase 7, caspase 3, PARP and β-actin, all antibodies were diluted by 1:1000) overnight at 4 °C. The membrane was washed and incubated with the Dylight 800-labeled goat anti-rabbit fluorescence secondary antibody for 1 h. The membrane was scanned by the Odyssey infrared imaging system (LI-COR Biosciences, Nebraska, USA).

### Quantitative polymerase chain reaction (Q-PCR)

BGC-823 cells were seeded in 6-well plates at the density of 3 × 10^5^ cells/well in 2 mL medium. After cells were cultured to 60~70% confluence/well, cells were treated by costunolide with different concentration (0, 5, 10, 15, 20 and 25 μM) for 24 h. Cells were washed with PBS twice and cell total RNA was extracted using the TRIzol. mRNA was transcribed to cDNA by using the Reverse Transcription PCR kit. The specific gene mRNA relative expression was detected by using the Quantitative-PCR kit and fluorescence quantitative PCR equipment (Applied Biosystems, California, USA). Data was analyzed by the 2^-∆∆Ct^ method. GAPDH gene is the reference gene. Q-PCR primers were synthesized by GenScript Biotech (Nanjing, China) and are listed in Table 1.

**Table 1:** Primers of Q-PCR

Gene	Primer	Sequence (5′-3′)	Product (bp)
GAPDH	Forward Reverse	GCAAATTCCATGGCACCGTC GACTCCACGACGTACTCAGC	133
Bax	Forward Reverse	GAACCATCATGGGCTGGACA GCGTCCCAAAGTAGGAGAGG	102
Bcl-2	Forward Reverse	GAACTGGGGGAGGATTGTGG CCGTACAGTTCCACAAAGGC	183
puma	Forward Reverse	GGAGACAAGAGGAGCAGCAG GGTAAGGGCAGGAGTCCCAT	82
Bak1	Forward Reverse	CACAGAGGAGGTTTTCCGCA ATAGCGTCGGTTGATGTCGT	178

### Nude mice xenograft experiment

The gastric cancer BGC-823 cells (2 × 10^6^ cells in 0.2 mL PBS) was injected subcutaneously into the right flank of athymic BALB/c nude mice. After 12 days, 16 mice were randomly divided into 4 groups: blank (saline), DMSO (saline containing 1% DMSO), cisplatin (2 mg/kg, resolved in saline) and costunolide (50 mg/kg, resolved in saline containing 1% DMSO) group. The mice of blank, DMSO, and costunolide group were gavage administrated every two day until the end of animal experiment. The mice of cisplatin group were injected intraperitoneally every three day and administrated 3 times in total. Tumor treatment in animals lasted for 24 days. Body weight and tumor size were measured every three day. Formula of tumor size: 0.5 × length×width^2^. Apoptosis of gastric tumor was detected by Tunel assay. Apoptosis related proteins (Bax, Bcl-2, cleaved caspase 9, cleaved caspase 3, β-actin is internal reference protein) of gastric tumor was detected by WB. Apoptosis related genes (puma, Bax and Bcl-2, GAPDH is internal reference gene) expression of gastric tumor was detected by Q-PCR.

### Tunel assay

The tumor tissue was fixed with 4% maldehyde for 24 h, dehydrated and embedded with paraffin. Tumor tissue was cut into 5 μm wax pieces. The tissue was dewaxed and hydrated. The tissue was treated by protein kinase K and 3% H_2_O_2_. The sample was incubated with 50 μL Tunel detection solution (the component of Tunel staining kit) for 1 h at 37 °C and then incubated Streptavidin-HRP working solution. The DAB solution was added to tissue to colorating the place of apoptosis. The sample was washed twice and observed by light inverted microscope (Olympus, Tokyo, Japan).

### Statistical analysis

Data are expressed as the mean ± standard deviation. The difference between different groups was analyzed using one-way analysis of variance (with Tukey’s post-hoc test) or Student’s t-test. *P < 0.05 and **P < 0.01 was considered to indicate a statistically significant difference. The data were analyzed using SPSS software (IBM SPSS Inc., New York, USA) and graphs were plotted using GraphPad Prism software (GraphPad Software Inc., California, USA).

## Results

### Costunolide inhibited the viability of gastric adenocarcinoma BGC-823 cells

The purity of costunolide (compound formula: C_15_H_20_O_2_, molecular weight: 232.32, CAS No.: 553–21-9) was measured by HPLC in order to ensure the quality and accuracy of experiments. The HPLC analysis showed that the purity of costunolide is 99.87% (Fig. 1b). The viability of BGC-823 cells treated by costunolide with different concentration (0, 5, 10, 15, 20, 25, 30, 35 and 40 μM) is detected by MTT assay at 24 and 48 h. MTT assay indicated that costunolide inhibited the viability of BGC-823 cells significantly in a concentration and time dependent manner (Fig. 1c). The IC50 value of costunolide at 24 and 48 h is 32.80 and 23.12 μM respectively. The morphology of BGC-823 cells treated by costunolide with different concentration (0, 5, 10, 15, 20 and 25 μM) for 24 and 48 h was observed by light inverted microscopy. Costunolide caused the shrinkage and deformation of BGC-823 cells obviously (Fig. 1d).

### Costunolide induced the apoptosis of gastric adenocarcinoma BGC-823 cells

**Fig. 2:**
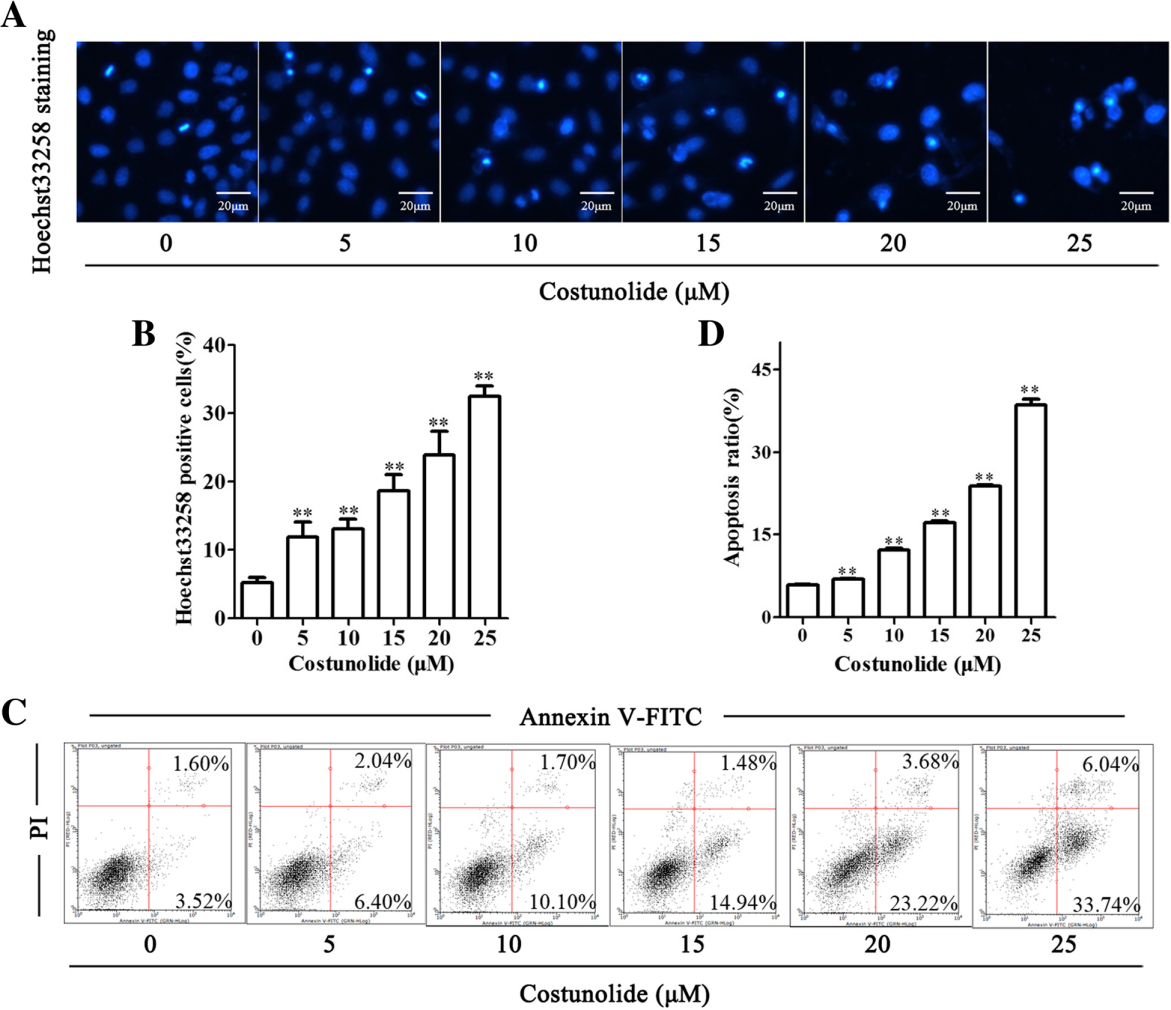
Costunolide induced the apoptosis of gastric adenocarcinoma BGC-823 cells. **a** Hoechst33258 staining of BGC-823 cells treated by costunolide with different concentration (0, 5, 10, 15, 20 and 25 μM) for 24 h are photographed by fluorescence microscope (magnification: 200×, bar in the pictures represents 20 μm). **b** Statistical data of Hoechst33258 positive cells. **c** Apoptosis of BGC-823 cells treated by costunolide with different concentration (0, 5, 10, 15, 20 and 25 μM) for 24 h and stained by Annexin V-FITC and PI is detected by flow cytometry. **d** Statistical data of BGC-823 cell apoptosis. Compared to control group, *P < 0.05, **P < 0.01

According to the results of MTT assay, costunolide inhibited the viability of gastric cancer cells, we further study whether costunolide induced the apoptosis of BGC-823 cells. BGC-823 cells treated by costunolide with different concentration (0, 5, 10, 15, 20 and 25 μM) for 24 h were stained by Hoechst33258 and photographed by invert fluorescence microscope. Also, apoptosis of BGC-823 cells treated by costunolide and stained by Annexin V-FITC and PI is detected by flow cytometry. Hoechst33258 staining results and flow cytometry analysis showed that costunolide induced apoptosis of gastric cells significantly (Fig. 2).

### Costunolide changed the ΔΨm and induced mitochondria-mediated apoptosis in gastric adenocarcinoma cells

**Fig. 3:**
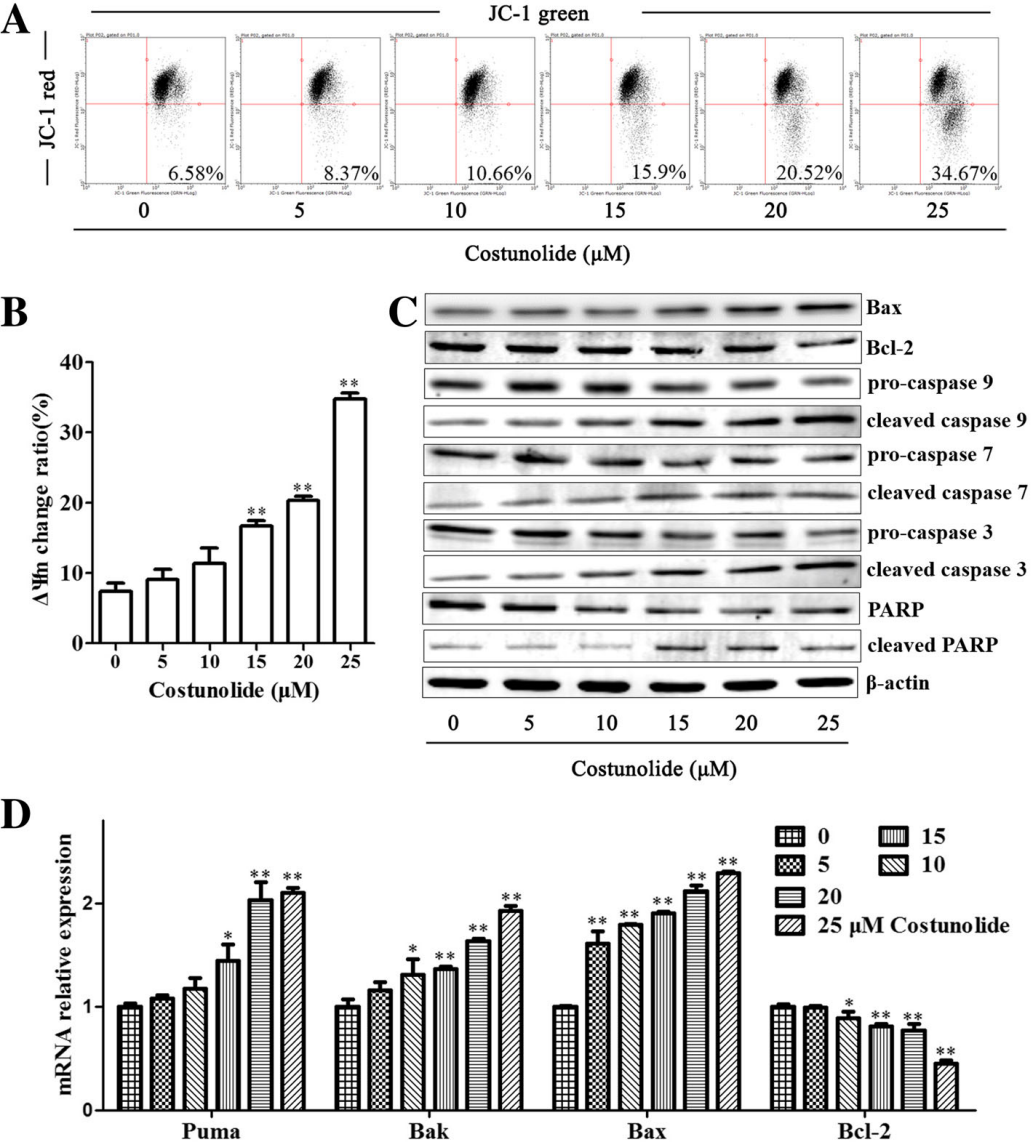
Costunolide changed the ΔΨm and induced mitochondria-mediated apoptosis in gastric adenocarcinoma cells. **a** ΔΨm of BGC-823 cells treated by costunolide with different concentration (0, 5, 10, 15, 20 and 25 μM) for 24 h and stained by JC-1 is detected by flow cytometry. **b** Statistical data of ΔΨm change ratio. **c** Apoptosis related proteins (Bax, Bcl-2, pro-caspase 9, cleaved caspase 9, pro-caspase 7, cleaved caspase 7, pro-caspase 3, cleaved caspase 3, PARP and cleaved PARP, β-actin is internal reference protein) of BGC-823 cells treated by costunolide with different concentration (0, 5, 10, 15, 20 and 25 μM) for 24 h is detected by WB. **d** Apoptosis related genes (puma, Bak1, Bax and Bcl-2, GAPDH is internal reference gene) expression of BGC-823 cells treated by costunolide with different concentration (0, 5, 10, 15, 20 and 25 μM) for 24 h is detected by Q-PCR. Compared to control group, *P < 0.05, **P < 0.01

The alteration of ΔΨm is closely related to the apoptosis, especially the early apoptosis of cancer cells [6]. JC-1 is a probe of ΔΨm. When the potential is normal, JC-1 forms aggregates that exhibit red fluorescence. When the potential drops, JC-1 exists as a monomer at low concentrations and yields green fluorescence [18]. Thence, in this research, BGC-823 cells treated by costunolide with different concentration (0, 5, 10, 15, 20 and 25 μM) for 24 h and stained by JC-1. Cells were detected by flow cytometry. The flow cytometry analysis indicated that costunolide led to the depolarization of ΔΨm of gastric cancer cells in a concentration dependent manner (Fig. 3a and b).

The mechanisms of apoptosis and change of ΔΨm is required to further investigate. The ΔΨm change and apoptosis induced by costunolide may be associated to many related proteins and genes [6]. Apoptosis related proteins (Bax, Bcl-2, pro-caspase 9, cleaved caspase 9, pro-caspase 7, cleaved caspase 7, pro-caspase 3, cleaved caspase 3, PARP and cleaved PARP) and genes (puma, Bak1, Bax and Bcl-2) of BGC-823 cells treated by costunolide with different concentration (0, 5, 10, 15, 20 and 25 μM) for 24 h was detected by WB and Q-PCR respectively. WB analysis showed that costunolide increased the expression of Bax, cleaved caspase 9, cleaved caspase 7, cleaved caspase 3 and cleaved PARP proteins and decreased the expression of Bcl-2, pro-caspase 9, pro-caspase 7, pro-caspase 3 and PARP proteins (Fig. 3c). Q-PCR analysis indicated that costunolide upregulated the expression of puma, Bak1 and Bax mRNA and downregulated the expression of Bcl-2 mRNA (Fig. 3d). Costunolide induced mitochondria-mediated apoptosis in gastric cancer cells.

### Costunolide inhibited the growth and induced mitochondria-mediated apoptosis of gastric tumor in xenografted nude mice

**Fig. 4:**
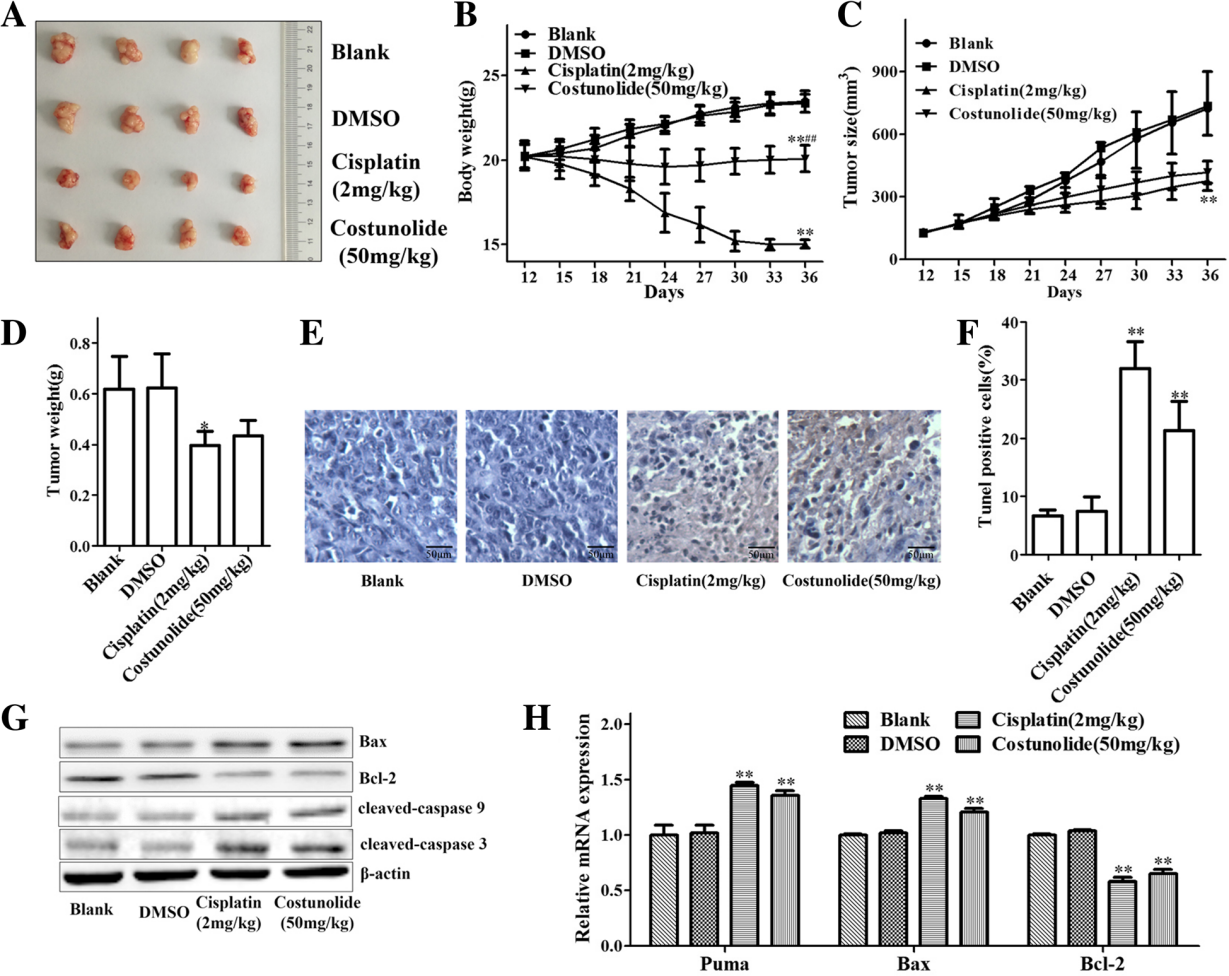
Costunolide inhibited the growth and induced mitochondria-mediated apoptosis of gastric tumor in xenografted nude mice. **a** Photograph of gastric tumor is taken after sacrificing mice. There are 4 groups: blank, DMSO, cisplatin (2 mg/kg) and costunolide (50 mg/kg) group. **b** Body weight of mice was measured every three day. **c** Tumor size of mice was measured by vernier caliper every three day. **d** Tumor weight of mice was measured after sacrificing mice. **e** Tunel staining of gastric tumor. **f** Statistical data of Tunel staining positive cells. **g** Apoptosis related proteins (Bax, Bcl-2, cleaved caspase 9, cleaved caspase 3, β-actin is internal reference protein) of gastric tumor of nude mice is detected by WB. **h** Apoptosis related genes (puma, Bax and Bcl-2, GAPDH is internal reference gene) expression of gastric tumor is detected by Q-PCR. Compared to control group, *P < 0.05, **P < 0.01. Compared to cisplatin group, ^#^*P* < 0.05, ^##^*P* < 0.01

To evaluate the inhibitory effect of costunolide on gastric cancer in vivo, the gastric cancer xenografts in nude mice were established. The experimental data in vivo indicated that costunolide decreased the tumor volume and weight of gastric cancer tumors (Fig. 4a, c and d). Compare to cisplatin group, costunolide improved the weight of nude mice (Fig. 4b). These data demonstrated that costunolide significantly inhibited the growth of gastric cancer xenografts in nude mice.

**Fig. 5:**
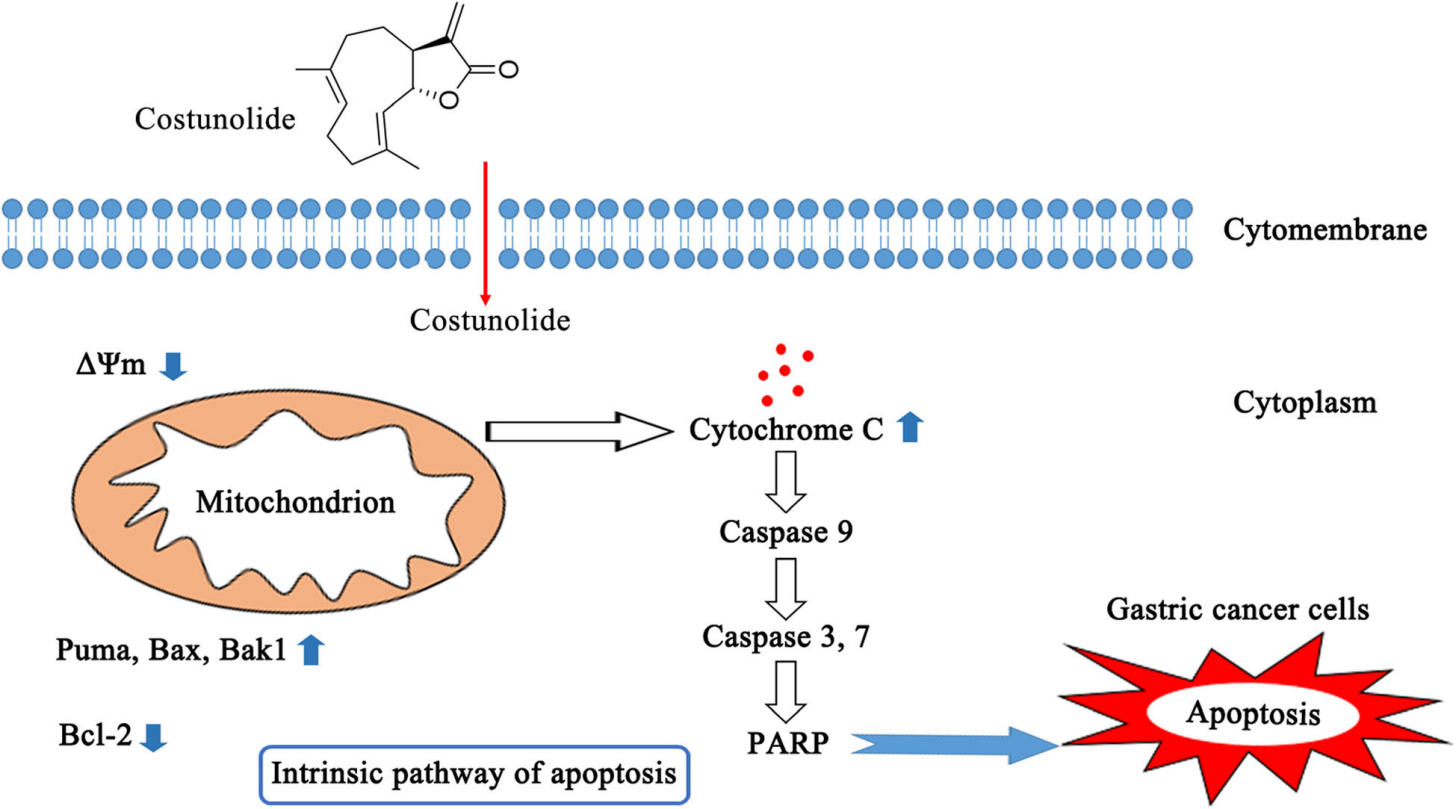
Mechanisms of mitochondria-mediated apoptosis induced by costunolide in human gastric adenocarcinoma BGC-823 cells

Apoptosis of gastric tumor was detected by Tunel assay. The results indicated that costunolide induced apoptosis of gastric tumor (Fig. 5e and f). WB analysis showed that costunolide increased the expression of cleaved caspase 9, cleaved caspase 3 and Bax proteins and decreased the expression of Bcl-2 protein in xenografted tumor (Fig. 5g). Q-PCR analysis indicated that costunolide upregulated the expression of puma and Bax mRNA and downregulated the expression of Bcl-2 mRNA in xenografted tumor (Fig. 5h). The results suggested that costunolide induced apoptosis of gastric tumor obviously in vivo.

## Discussion

GC is a kind of digest system malignancy and remains difficult to treat and prevent in clinical practice so far [1, 2]. Therefore, looking for the effective drugs and studying the underlying mechanisms are necessary and meaningful. Natural products from plants are the important source of drugs to treat or prevent GC. Costunolide, a sesquiterpene lactone compound, is mainly extracted from and an effective constituent of *Radix Aucklandiae*. Costunolide is also derived from *Laurus nobilis*, *Magnolia grandiflora*, and so on. It was reported that costunolide exhibits various pharmacological properties and inhibits many cancers [7]. However, the study on the effect of costunolide on GC and the mechanisms is still scarce up to now. In this study, we have researched the effect of costunolide on GC and the pharmacological mechanisms in vitro and in vivo.

According to the results of HPLC, the purity of costunolide is 99.87% (Fig. 1b), which ensured the quality and accuracy of pharmaceutical research. Apoptosis is closely related to the proliferative activity and tumorigenesis of gastric cancer. Apoptosis could be the target of drugs. Tumor cell apoptosis can be used as a target for research drugs [5]. Costunolide significantly inhibited the viability of BGC-823 cells and showed the concentration dependent manner (Fig. 1c). The IC50 value of costunolide at 24 and 48 h is 32.80 and 23.12 μM respectively. Meanwhile, costunolide led to cell irregularity, float and death (Fig. 1d). It has been demonstrated that costunolide induces apoptosis of a variety of tumor cells, such as colon cancer cells, prostate cancer cells, lung squamous carcinoma cells, hepatocellular carcinoma cells, bladder cancer cells, ovarian cancer cells and breast cancer cells [9–12, 14, 19]. However, whether the costunolide can cause apoptosis of gastric cancer cells is less studied. In our research, flow cytometry analysis and Hoechst33258 staining results indicated that costunolide obviously induced the apoptosis of BGC-823 cells (Fig. 2a). The mechanism of apoptosis induced by costunolide is further explored.

It was reported that apoptosis pathways consist of the extrinsic pathway, the intrinsic pathway (also called mitochondrial-mediated apoptotic pathway), and endoplasmic reticulum stress pathway [5, 12]. Mitochondrial pathway apoptosis is the critical and common way of apoptosis induced by chemotherapeutic drugs, oxidative stress, UV and so on [20]. The mitochondrial-mediated apoptotic pathway is the important target of treatment for GC and closely related to alteration of ΔΨm. The change of ΔΨm increases the permeability of mitochondrial membrane and can leads to release of cytochrome C to cytoplasm. The complex formed by cytochrome C and other protein factors induces activation of caspase 9 [6]. Costunolide changed the ΔΨm of gastric cancer cells significantly according to the results of JC-1 staining (Fig. 3a).

The change of ΔΨm is the early event of mitochondrial pathway apoptosis. Apoptosis is associated with the many proteins and genes closely. It has been demonstrated that imbalance of Bcl-2 and Bax can induce the ΔΨm change and apoptosis of cancer cells [6]. To further research the underlying mechanisms of mitochondrial pathway apoptosis induced by costunolide in BGC-823 cells, the apoptosis related proteins and genes were detected. Costunolide increased the expression of Bax, cleaved caspase 9, cleaved caspase 7, cleaved caspase 3 and cleaved PARP proteins and decreased the expression of Bcl-2 and PARP proteins (Fig. 3c). Costunolide upregulated the expression of puma, Bak1 and Bax mRNA and downregulated the expression of Bcl-2 mRNA (Fig. 3d). The Bcl-2 family contains of anti-apoptosis and pro-apoptosis proteins. The pro-apoptosis proteins, such as Bax, Bak and Bid, promote apoptosis. The anti-apoptosis proteins, such as Bcl-2 and Bcl-xL. Caspase 3 and 7 are the downstream proteins of apoptosis pathway and the executioner caspases [20].

The tumor xenografted nude mice model is a method to evaluate the anti-tumor effect of the drug in vivo. It was reported that costunolide induced apoptosis of platinum-resistant cells suppressed tumor growth in human ovarian cancer cells bearing mouse model [17]. In this study, the BGC-823 cells xenografted nude mice model was established and used to study the pharmacological mechanisms of gastric cancer inhibited by costunolide. According to the statistics of animal experiments, costunolide inhibited tumor and induced apoptosis of gastric tumor (Fig. 4b-e). We further researched the change of proteins and genes related to mitochondrial-mediated apoptosis in xenografted nude mice model. Costunolide increased the expression of cleaved caspase 9, cleaved caspase 3 and Bax proteins and decreased the expression of Bcl-2 protein in xenografted tumor (Fig. 4g). Costunolide upregulated the expression of puma and Bax mRNA and decreased the expression of Bcl-2 mRNA in xenografted tumor (Fig. 4h). Those results indicated that costunolide activated the mitochondrial-mediated apoptotic pathway of gastric cancer in vivo.

## Conclusions

Collectively, costunolide inhibited the viability and induced the apoptosis of human gastric adenocarcinoma BGC-823 cells in vitro and in vivo via activating the mitochondrial pathway (Fig. 5). However, there are still many unresolved problems about the pharmacological mechanisms of costunolide. Reactive oxygen species (ROS) production induced by costunolide may be the important intermediate link of mitochondrial apoptosis. Costunolide has the potential to activate extrinsic pathway or endoplasmic reticulum stress pathway [21–23]. Our research group will be further to explore the mechanisms of costunolide via signaling pathway (such as p53, NF-κB and Wnt/β-catenin signaling pathway) and even find the specific molecular target [6, 7]. This research suggested that costunolide could be the candidate and therapeutic agent for treatment of GC.

## Data Availability

The datasets used and analysed during the current study are available from the corresponding author on reasonable request.

## Abbreviations

Bak1: Bcl-2 antagonist/killer 1
Bax: Bcl-2-associated X protein
Bcl-2: B-cell lymphoma-2
GC: gastric cancer
MTT: 3-(4,5-Dimethyl-2-thiazolyl)-2,5-diphenyl-2H-tetrazolium bromide
PARP: poly ADP ribose polymerase
Puma: p53 upregulated modulator of apoptosis
ΔΨm: mitochondrial membrane potential
